# Insight into the immunomodulatory and chemotherapeutic mechanisms of paeonol (Review)

**DOI:** 10.3892/mi.2025.223

**Published:** 2025-02-26

**Authors:** Precious Barnes, Elvis Agbo, Faustina Halm-Lai, Kwabena Dankwa, Roland Osei Saahene, Samuel Victor Nuvor, Dorcas Obiri-Yeboah, Ewura Seidu Yahaya

**Affiliations:** 1 Department of Chemical Pathology, School of Medical Sciences, College of Health and Allied Sciences, University of Cape Coast, Cape Coast 00233, Ghana; 2 Department of Human Anatomy, Histology and Embryology, College of Medicine, Jinggangshan University, Ji'an, Jiangxi 343000, P.R. China; 3 Department of Microbiology and Immunology, School of Medical Sciences, College of Health and Allied Sciences, University of Cape Coast, Cape Coast, 00233, Ghana; 4 Department of Pharmacology, School of Medical Sciences, College of Health and Allied Sciences, University of Cape Coast, Cape Coast 00233, Ghana

**Keywords:** paeonol, immunomodulator, anticancer agent, hepatoprotective effects, neuroprotective effects

## Abstract

Paeonol a, pharmacologically active constituent obtained from the root bark of *Paeonia suffruticosa* has been extensively utilized as a traditional Chinese medicine for the treatment, prevention and control of several diseases for years. Paeonol has been reported to possess key immunomodulatory properties; however, the underlying mechanisms involved in its immunomodulatory and anticancer effects have not been extensively researched due to limitations in terms of design, conduct and interpretation. The present review focuses on both the *in vitro* and *in vivo* immunosuppressive and anticancer effects of paeonol and the underlying mechanisms of action. The present literature review aimed to include all the notable findings published on Google Scholar, PubMed, Web of Science, SciFinder and ScienceDirect. Overall, paeonol possesses multifaceted pharmacological activities with potential for use in the development of novel immunomodulator and anticancer therapeutic agents. Paeonol decreases IL-1β expression to repress several inflammatory mediators, such as NO, iNOS, COX2 and PEG2 in the inhibition of the NLRP3 inflammasome, NF-κB, MAPK and TLR4 pathways to provide multiple levels immunosuppression; these effects may be beneficial in immune-related diseases. Furthermore, paeonol inhibits cancer cell growth, proliferation, invasion and metastasis by inducing cell apoptosis and the suppression of the TLR4/NF-κB/STAT3/MAPK/PI3K/AKT/CHOP/VEGF/HIF-1α, pathways. The present review aimed to promote further research to exploit the potential use of paeonol as a novel therapeutic agent for immunomodulation and cancer management.

## 1. Introduction

The immune system is comprised of white blood cells (monocytes, macrophages, dendritic cells, neutrophils, eosinophils, basophils and lymphocytes) and special immune molecules (cytokines, antibodies, complement proteins and signaling pathways) that have evolved to resist infections (1). The immune system is classified into two broad categories: The innate and adaptive immune system, which collaborate to protect the body against foreign invaders and aid in the maintenance of the normal homeostasis of the body. Innate immunity is a non-specific immune system which provides immediate initial defense against infections (2), while adaptive immunity is a specific immune system which develops more slowly and provides specific and effective defense against invading pathogens (3). Several intrinsic and extrinsic agents can affect the functioning of the immune system, rendering it either immunostimulatory or immunosuppressive (3). Immunomodulation is the alteration of the responsiveness of the body's immune system caused by a chemical substance that can suppress or activate its function in the fight against diseases (4). A malfunctioning or imbalanced immune system can result in the development of a variety of chronic diseases, such as cancer, autoimmune disorders, allergies, viral infections, inflammatory bowel diseases and numerous others (5). Immunomodulators are bioactive agents that can regulate immunological events, such as stimulating phagocytes, natural killer cell, and activating T- and B-cells (6). The organic entity of natural or artificial sources with the characteristics of inhibiting, stimulating or modulating the constituents of either non-specific or specific immunity are also referred to as immune modulators or biological response modifiers. Generally, in medical practice, these sources are grouped as immunosuppressant, immunostimulant and immunoadjuvant (7,8). Advancements in immunological research indicate that the suppression of the immune system due to stressful environmental conditions can result in the development of a variety of diseases (9). Therefore, it is evident that immunomodulators can serve as alternative therapies for the management of various diseases, such as activating immune responsiveness impairment by HIV (10,11) or suppressing it during transplantation (12). Additionally, the application of these modulators in cancer treatment is crucial due to their central role in supporting immune responses (13). As biological response modifiers, immunomodulatory agents inhibit tumor cells directly or by enhancing host defense mechanism such as induction of apoptosis against the tumor (14). The immunological effects of immunomodulators can be evaluated by their specified actions on immune cells, effector mechanisms, inhibition of nitric oxide (NO) and reactive oxygen species (ROS) production, the secretion of inflammatory cytokines, signaling pathways in macrophage cells and phagocytosis activity (15).

Cortex moutan, the root bark of *Paeonia suffruticosa* has been extensively utilized as a traditional Chinese medicine for the prevention and control of several diseases for years with a good safety and efficacy profile (16). Paeonol is a pharmacologically effective phenolic constituent isolated from Cortex Moutan. Paeonol has been reported to possess extensive therapeutic effects on the innate and adaptive immune system in several human diseases (17-21) and cancers (22-24), which are associated with inhibition of immune signaling pathways (25-27), NO (21) and ROS production (28), inflammatory cytokines (29,30), immune cells (20,31), phagocytes (17,32) and the induction of apoptosis (14,25,33). Paeonol has been applied together with other drugs to effectively protect against oropharyngeal candidiasis (34), hepatotoxicity (35) and cardiotoxicity (36), indicating that paeonol can potentially act as an alternative or complementary medicine to offset deficiencies and unresolved safety of current drugs for treatment of diseases. In the light of the therapeutic prospects and pharmacological activities of paeonol, the present review provides an overview of the underlying mechanisms of paeonol in immunomodulation and chemoprevention.

## 2. Literature search methods

To assess the existing studies on the immunomodulatory and chemotherapeutic potentials of paeonol for a wide range of human diseases, the Pubmed, Medline, Google Scholar and Web of Science, SciFinder and Science Direct databases were used to search for literature published from 2013 up to 2024. Paeonol and immunomodulation or cancer were the key words used for the search. All relevant full-text research articles with titles/abstract were incorporated without language restrictions. Citations from selected publications were tracked and potential papers added.

## 3. Botanical characteristics of *Paeonia suffruticosa*

Peonies mainly thrive well in northwest Africa, temperate Eurasia and western North America. Nevertheless, wild peony is endemic to China, where it was initially domesticated (37). It is a perennial shrub with brown-gray stems and grows to a height of 1.5 m. It has long oval leaflets, solitary, single or double flowers with an asymmetrical apex. The bark of the root is yellow to brown with lignified fleshy center and a completely developed root system (38-40). It has a tube-shaped bark, with breaks along the entire length. The bark is slightly curly inward, 3-8 cm long, 0.5-1.2 cm in diameter and 0.1-0.4 cm thick. The external surface of the bark is reddish-brown or grayish-brown. The cork drop is pink with projecting pore spaces, light brown or grayish-yellow inner surface with a fine longitudinal texture and sparkling crystals (16).

## 4. Paeonol: The principal active ingredient of *Paeonia suffruticos*a

Cortex moutan has several bioactive constituents, such as gallic acid derivatives, flavonoids, triterpenes, monoterpene glycosides, and in particular, phenolic compounds. The principal active elements of Cortex moutan comprise, paeoniflorin, gallic acid, 1,2,3,4,6-penta-*O*-gallic acid-β-D-glucose and the phenolic compound paeonol (16). The active ingredients have been reported to be extracted using methods, such as organic solvent extraction, ultrasonic assisted extraction, steam distillation, and CO_2_ supercritical fluid extraction (41-43).

## 5. Immunomodulatory effects of paeonol

*In vitro and in vivo immunosuppressive effects of paeonol*. Diverse *in vitro* and *in vivo* research exploiting paeonol has indicated its potential immunosuppressive activities. Paeonol administration was previously shown to inhibit inflammation by downregulating lipase, amylase, IL-1β and IL-6. Moreover, the drug decreased ROS, restored mitochondrial membrane potential and suppressed M1 macrophage polarization through the NOD-like receptor protein 3 (NLRP3) pathway in bone marrow-derived macrophages (21). In another study, paeonol improves fluconazole or amphotericin B application during the treatment of EC109 cells and in a mouse model of oropharyngeal candidiasis by suppressing HIF-1α, IL-17A and IL-23 expression at the mRNA and protein level (34). A previous study also demonstrates that out of seven components of Cortex moutan Radicis studied in the treatment of antibiotic-associated diarrhea in mice and RAW 264.7 cells, paeonol attenuated NO production and IL-6 expression, decreased the level of TNF-α in a concentration-dependent manner, as well as the mRNA levels of IL-6, IL-1β and TNF-α (30). Also, COX-2 and NF-κB levels decreased as confirmed by western blot analysis; however, at the concentration of 25 µM, the p-p38 MAPK level was upregulated and was downregulated at 50 µM. Furthermore, compared with the control group, the TNF-α and IL-4 levels were decreased (30). As previously reported, Miao *et al* (32) indicated that paeonol improved the phagocytic capacity of macrophages by restricting high-mobility group box 1 to the nucleus. Additionally, it promoted the phosphorylation of focal adhesion kinase to stimulate the development of pseudopod in enhancing phagocytosis (32). Paeonol has been documented to suppress the miR-155/JAK1-STAT1 pathway to inhibit lipopolysaccharide (LPS) and IFN-γ-induced macrophage M1 polarization via the downregulation of F4/80 and CD86, IL-6 and TNF-α in mice and RAW264.7 cells (44). A previous study demonstrated that the oral administration of paeonol suppressed the development of 1-chloro-2,4-dinitrobenzene-induced AD-like lesions in BALB/c mice by downregulating the lesion severity, epidermal thickness and infiltration of mast cells (31). Moreover, this led to decreased levels of immunoglobulin E and IL-4, histamine, IL-13, IL-31 and thymic stromal lymphopoietin, together with the modulation of the T-helper (Th1/Th2) ratio. Paeonol regulated the (Th) cell subset (Th1/Th2) ratio by downregulating immunoglobulin E, IL-4, histamine, IL-13, IL-31 and thymic stromal lymphopoietin levels. Furthermore, its application attenuated phosphorylated (p)-p38 and p-ERK. In addition, the production of TNF-α and histamine was decreased, and p38/ERK/MAPK signaling inhibited in P815 cells. The aforementioned demonstrates that the drug act on cluster of differentiation 4+ T-and mast cells to inhibit allergic inflammatory conditions (31). In another report, paeonol suppressed NF-κB, IL-6 and TNF-α through denosine 5'-monophosphate-activated protein kinase (AMPK) and the glycogen synthase kinase-3 (GSK-3) pathway to prevent inflammation (29). Liu *et al* (45) investigated the effects of paeonol on THP-1 cells and human umbilical vein endothelial cells (HUVECs). The authors concluded that the drug upregulated miR-223 in THP-1 extracted exosomes and in HUVECs following the intake of exosomes, but reduced STAT3 and p-STAT3 expression in HUVECs. In addition, IL-1β, IL-6, ICAM-1 and VCAM-1 levels were downregulated in HUVECs, and THP-1 cell adhesion to HUVECs was attenuated to reduce the inflammatory response (45). Another study demonstrated that paeonol, at various concentrations, exerted anti-inflammatory effects against IL-1β-induced inflammation by suppressing NO and prostaglandin E2 (PGE2) production (46). Additionally, that study revealed that inducible nitric oxide synthase (iNOS), COX-2, MMP-1, MMP-3 and MMP-13 overexpression were reversed together with inhibition of NF-κB, PI3K and AKT activated signals (46). In a study investigating the effects of paeonol in a mouse model of imiquimod-induced psoriasis-like skin lesions and murine bone marrow-derived dendritic cells stimulated by R848, it was demonstrated that the drug inhibited IL-23 and downregulated dendritic cells expressing MHCII, CD80 and CD86 *in vitro* (17). This indicated that paeonol suppressed the maturation and activation of dendritic cells by downregulating MyD88 and Toll-like receptor (TLR)8 proteins (17). Another study demonstrated that paeonol, in a dose-dependent manner, attenuated LPS-stimulated inflammation to protect kidney injury (18). That study employed ELISA, western blot analysis and immunohistochemistry to determine the expression of inflammatory cytokines, TLR4-NF-κB pathway, and phospho-NF-κB p65, respectively. The results revealed that paeonol repressed pro-inflammatory cytokines and upregulated anti-inflammatory cytokines. Furthermore, the p-IκBα and p-IKKβ, NF-κB p65, and NF-κB p65 DNA-binding activities were inhibited. In addition, it suppressed the TLR4-NF-κB signaling pathway (18). Paeonol has been demonstrated to suppress the PI3K/Akt/NF-κB pathway to reduce hepatotoxicity (35). As previously demonstrated, pre-treatment of BV-2 cells and mice with paeonol revealed its ability to regulate p-AMPK-α and GSK 3α/β to suppress the expression of NO, iNOS, COX-2 and ROS induced by LPS/INF-γ. Furthermore, the drug suppressed STAT3 and p38 pathways (47). In another study, the administration of paeonol suppressed IL-8 via its antioxidant activity to inhibit MAPKs/NF-κB signaling to alleviate inflammation induced by chronic cigarette smoke (26). Chen *et al* (48) reported that paeonol, in a concentration-dependent manner, downregulated the release of TNF-α, IL-1β, IL-6, and increased IL-10 in LPS-induced RAW 264.7 via the deactivation of IκBα, ERK1/2, JNK and p38 MAPK. The *in vitro* and *in vivo* mechanisms of action of paeonol reported above are presented in Table I.

In summary, a critical analysis of both *in vitro* and *in vivo* immunomodulatory mechanisms demonstrated that paeonol suppresses the discharge of pro-inflammatory cytokines, including IL-1β, IL-6, TNF-α and IL-8, while upregulating the anti-inflammatory cytokine, IL-10, in the treatment of several inflammation in various models. However, the specific genomic and proteomic changes induced by paeonol remain underexplored. Additionally, paeonol regulates the function of macrophages in promoting tissue repair by modulating polarization to an M2 anti-inflammatory phenotype while inhibiting M1. It promotes the maturation and activation of dendritic cells to initiate T-cell adaptive immunity. The drug mitigates allergy by decreasing immunoglobulin E, histamine, hence can be used to manage conditions such as asthma, atopic dermatitis and allergic rhinitis. Moreover, paeonol protects against kidney injury and hepatotoxicity by inhibiting oxidative stress, NF-κB and MAPK pathways, and decreases the generation of ROS. Furthermore, it modulates the NF-κB, PI3K/Akt, AMPK and MAPK pathways to exert multiple level immunosuppressive effects to attenuate inflammation extensively. In addition, it exerts synergistic effects when combined with conventional antifungal agents or anti-inflammatory drugs.

### In vitro immunosuppressive effects of paeonol

Recently, studies have demonstrated that paeonol promotes M2 macrophage polarization by suppressing M1 polarization (49,50) through the inhibition of the NF-κB and MAPK pathways (49). Miao *et al* (51) demonstrated that the promotion of nuclear p53 by paeonol served as a transcript and increased triggering receptor expressed on myeloid cells-2 to activate macrophage lipid metabolic capacity and phagocytic function. In another study, paeonol downregulated IL-1β and caspase-1 level, attenuated the monosodium urate (MSU)-induced interaction of pro-caspase-1 and apoptosis-associated speck-like protein containing caspase recruitment domain (ASC) in J774A.1 cells induced by LPS plus MSU (52). The drug decreased IL-1β, NLRP3, p-IKK, p-IκBα, and p-p65 in J774A.1 cells induced by LPS alone but did not affect the ASC level. Moreover, the IκBα content was stimulated and a greater amount of cytoplasmic p65 retained. Furthermore, paeonol decreased p65 DNA-binding action and curtailed p-JNK, p-ERK and p-p38 level. The study concluded that the inhibition of the NLRP3 inflammasome, NF-κB and MAPK pathway activation by paeonol occurred through the suppression of IL-1β (52). Another study assessed the paeonol-mediated suppression of LPS-activated inflammation through the TLR4 signaling pathway in N9 microglia cells (53). That study evaluated NO, IL-1β and PGE2 using ELISA. Additionally, COX-2, iNOS, TLR4, MyD88, IRAK4, TNFR-associated factor 6 (TRAF6), p-IkB-α, and NF-kB p65, as well as p-P38, p-JNK and p-ERK were examined using western blot analysis. The results revealed that paeonol significantly repressed the production of pro-inflammatory products, including NO, IL-1β, and PGE2 as well as the expression of iNOS and COX-2. In addition, the TLR4, MyD88, IRAK4, TRAF6, p-IkB-α and NF-κB p65, as well as p-p38, p-JNK and p-ERK expression levels were significantly reduced (53). In another study, paeonol upregulated NO, iNOS and downregulated ROS (28). Moreover, the drug attenuated TNF-α, IL-1β, IL-6 and monocyte chemoattractant protein-1 (MCP-1) with a significant decrease in RAGE and CD36, and an increase in SR-A and SR-B1 levels. The authors of that study reported that the anti-inflammatory response was likely mediated via the RAGE, CD36, SR-A and SR-B1-pathways with NADPH oxidase-dependent ROS generation (28). The aforementioned *in vitro* studies have revealed that paeonol decreases IL-1β to repress several inflammatory mediators, such as NO, iNOS, COX2 and PEG2 in the inhibition of the NLRP3 inflammasome, and also inhibits the NF-κB, MAPK and TLR4 pathways to provide a broad-spectrum effect on immune-related diseases, to function as an anti-inflammatory agent.

### In vivo immunosuppressive effects of paeonol

**Figure 1:**
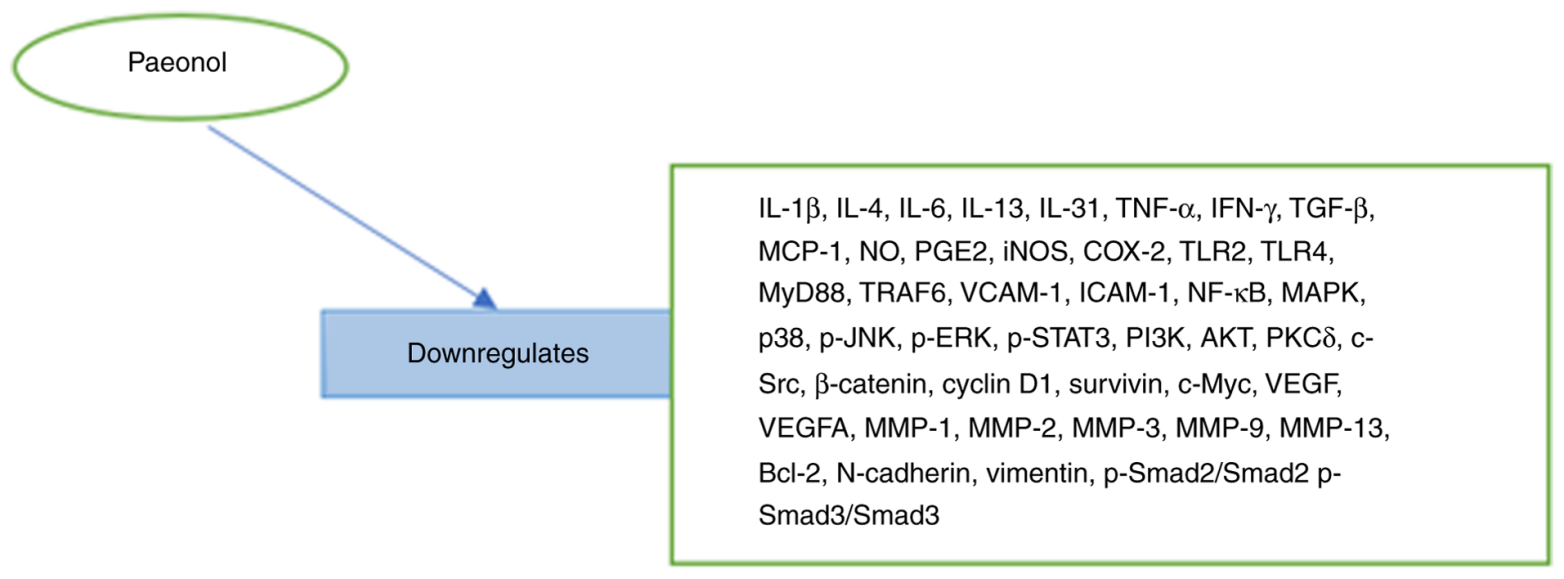
Key signaling pathways and mediators downregulated by paeonol described above. IL, interleukin; TNF-α tumor necrosis α; IFN-γ, interferon γ; TGF-β tumor growth factor β; MCP-1, monocyte chemoattractant protein; NO, nitric oxide; PGE2, prostaglandin E2; iNOS, inducible nitric oxide synthase; COX-2, cyclooxygenase 2; TLR, Toll-like receptor; MyD88, myeloid differentiation primary response 88; IRAK4, interleukin 1 receptor associated kinase 4; TRAF6, TNF receptor associated factor 6; VCAM-1, vascular cell adhesion molecule 1; ICAM-1, intercellular adhesion molecule 1; NF-κB, nuclear factor κB; MAPK, mitogen-activated protein kinase; p38, 38-kDa mitogen-activated protein kinase; p- JNK, phosphorylated Jun N-terminal kinase; p-ERK, phosphorylated extracellular signal-regulated kinase; STAT3, signal transducer and activator of transcription 3; P13K, phosphoinositide 3-kinase; AKT, serine/threonine kinase; PKCδ, protein kinase Cδ; VEGF, vascular endothelial growth factor; MMP, matrix metalloproteinase; Bcl-2, B cell lymphoma 2.

In a previous study, the administration of paeonol downregulated M1 polarization markers (IL1β, iNOS, CD32 and IL6), and upregulated the M2 polarization markers (IL10, CD206 and ARG-1) by decreasing iNOS, IL1β, RhoA and Rock1 in LPS-stimulated microglia (54). Additionally, during thermal hyperalgesia, the drug downregulated IL1β and IL8 and upregulated IL4 and TGF-β levels in the serum. It was revealed that in the spinal dorsal horn the drug downregulated IBA-1, IL1β, RhoA, RhoA-GTP, COX2, Rock1, and p-p38MAPK levels to reduce neuropathic pain (54). It has also been demonstrated that paeonol acts via the dectin-1/NF-κB pathway together with TLR2 and TLR4 to relieve fungal dysbiosis-associated ulcerative colitis (27). Recently, paeonol has been reported to significantly suppress NF-κB and MCP-1 *in vivo* (55). In another study, paeonol was shown to inhibit the inflammatory response by suppressing the mycobiota-mediated dectin-1/IL-1β signaling pathway (56). Paeonol administration has also been shown to attenuate liver injury and fibrosis. In a previous study, paeonol downregulated ALT and AST levels, and inhibited the TGF-b/Smad3 pathway by suppressing the activation of hepatic stellate cells to downregulate IL-6 and TNF-α, and reduced oxidative stress (57). Another study indicated that paeonol modulated nuclear factor E2-related factor 2 (Nrf2)/NF-κB/NFATc1 signaling in the inhibition of osteoclastogenesis to protect against periodontitis (58). It has been well-documented that paeonol improves the asthmatic condition by downregulating IFN-γ and upregulating IL-4 via the suppression of the TLR4/NF-κB and MAPK pathway (59). In another study, in LPS/D-galactosamine-induced acute liver failure, paeonol at 100 mg/kg markedly suppressed the iNOS, NO, COX-2 and PGE2 levels (60). The phosphorylation levels of IκB kinase (IKK), IκB and NF-κB (p65), which constitute the NF-κB pathway and MAPK signaling pathway molecules, including ERK, JNK and p38 were significantly inhibited. Moreover, the decreased expression of caspase-3, -8 and -9, and Bax, with the upregulated expression of Bcl-2 observed following paeonol treatment was attributed to the inhibition of hepatocyte apoptosis (60). Another study reported that paeonol at 200 and 400 mg/kg significantly decreased IL-17 and IL-6, and upregulated TGF-β1 expression in rats with 2,4,6-trinitrobenzenesulfonic acid-induced ulcerative colitis (61). In their study, Ding *et al* (62) investigated the anti-oxidative stress activity of paeonol against acetaminophen (APAP)-induced hepatotoxicity and indicated that it suppressed JNK phosphorylated protein. Moreover, the drug exerted a significant suppressive effect of H_2_O_2_ or APAP-induced ROS production. Furthermore, the levels of TNF-α, MCP-1, IL-1β and IL-6 were decreased in a concentration-dependent manner, while IKKα/β, IκBα and p65 phosphorylation were significantly inhibited (62). Paeonol has been documented to exert neuroprotective effects by repressing TLR-2 and TLR-4, Iba1, NF-κB (p50) and IL-1β, as well as by suppressing apoptosis in a Sprague-Dawley rat model of cerebral ischemia-reperfusion injury (63). Lee *et al* (64) evaluated the renal toxic effects of paeonol and reported that it downregulated TNF-α, IL-1β and NO, as well as decreased blood urea nitrogen and serum creatine levels (64). Key signaling pathways and mediators downregulated by paeonol *in vitro* or *in vivo* are presented in Fig. 1. The *in vitro* or *in vivo* mechanisms mentioned above are presented in Table II. In summary, paeonol has the capacity to be employed for the treatment of hepatotoxicity, acute liver diseases, liver failure, gastrointestinal disorders, ulcerative colitis, renal disorders and neurological conditions. However, further transcriptomics studies are required to elucidate its molecular mechanisms and maximize its therapeutic application in medical settings.

## 6. Clinical trials

A recent phase 2a randomized, placebo-controlled trial evaluated the efficacy and safety of a fixed-dose combination of apocynin (AP) and paeonol (PA) (APPA) in patients with symptomatic knee osteoarthritis (OA) (65). The study enrolled 152 participants with Kellgren-Lawrence grade 2-3 knee OA and moderate to severe pain (WOMAC pain ≥40/100). Over a period of 28 days, APPA at 800 mg twice daily demonstrated no statistically significant improvement in primary (WOMAC pain) or secondary outcomes (WOMAC function and total score) compared to the placebo. However, predefined subgroup analyses revealed a significant benefit of APPA in participants with nociplastic or neuropathic pain features. The treatment was well-tolerated with mild to moderate adverse events, primarily gastrointestinal. These findings highlight the safety profile of APPA and suggest its potential efficacy in specific OA subgroups, warranting further targeted research (65).

## 7. Chemopreventive potential of paeonol

The antitumor effects of paeonol on breast (14,24,36,66,67), bladder (68), colorectal (23,69), colon (70), gastric (22,71,72), lung (73-75), osteosarcoma (76), ovarian (77-81), prostate (82), chondrosarcoma (83), pancreatic (84), cervical (33,85), melanoma (25), renal (86), hepatocellular (87-89) and oral (90) cancers have been documented.

In a previous study, paeonol induced the apoptosis of breast cancer by regulating CXCL4/CXCR3-B signaling to downregulate heme oxygenase and Nrf2 to upregulate BACH1(14). In another study, paeonol and epirubicin synergistically inhibited tumor growth and promoted apoptosis via the activation of PARP, Bax and caspase-3 by suppressing p38/JNK/ERK MAPKs compared to epirubicin alone (36). Additionally, paeonol inhibited the NF-κB pathway to alleviate epirubicin-induced cardiotoxicity (36). Zhang *et al* (66) demonstrated that paeonol downregulated SET, protein phosphatase 2A and the PI3K/Akt pathway to prevent paclitaxel resistance in MCF-7/PTX cells. In a similar study, paeonol decreased P-glycoprotein, multidrug resistance associated protein 1, and breast cancer resistance protein to suppress transgelin 2-mediated paclitaxel resistance (67). In another study, the significant decrease in tumor weight observed by morphological analysis using hematoxylin and eosin staining, as well as TUNEL staining indicated that paeonol induced the apoptosis of tumor cells (24). Moreover, immunohistochemistry and western blot analysis demonstrated that the downregulation of Bcl-2 led to the upregulation of Bax, caspase-8 and caspase-3 to confirm the induction of apoptosis as the mechanism of inhibition employed by paeonol (24). In a recent study, the administration of paeonol exerted an inhibitory effect on cell proliferation and induced both *in vitro* and *in vivo* apoptosis by decreasing the Bcl-2/Bax ratio with the upregulation of caspase-3(68). The drug also suppressed the PI3K/AKT pathway in the inhibition of bladder cancer cell growth (68). In colorectal cancer (CRC), paeonol administration has been shown to induce apoptosis and arrest the cell cycle at the G0/G1-phase (69). Additionally, the drug downregulated β-catenin, cyclin D1, survivin and c-Myc in a concentration-dependent manner to suppress the Wnt/β-catenin signaling pathway (69). Li *et al* (23) investigated the effects of paeonol on CRC cells and revealed that paeonol suppressed cell proliferation by reducing COX-2 and PGE2 expression, and induced mitochondrial pathway apoptosis in a time-dependent manner. The apoptotic mechanism was associated with the upregulation of Bax and decreased Bcl-2 expression. Furthermore, the drug activated caspase-3 and caspase-9 and induced the loss of mitochondrial membrane potential (23). Flow cytometry analysis performed by Li *et al* (70) demonstrated that paeonol induced apoptosis and arrested the cell cycle at the G1 to S transition phase. Moreover, the treatment led to an upregulated intracellular calcium concentration and RUNX3 expression suggesting its antitumor mechanism (70). The administration of paeonol suppressed apatinib-resistant gastric cancer cell malignancy by downregulating LINC00665 and MAPK1, while upregulating miR-665(71). Paeonol has been shown to inhibit the NF-κB pathway by decreasing ERBB2 expression to suppress the proliferation of gastric cancer and induce apoptosis (72). The anticancer mechanism of paeonol has been shown to be associated with the downregulation of MMP-2 and-9 in a concentration-dependent manner to prevent invasion and migration (22). The anticancer effects of paeonol on lung cancer have been reported to be associated with the upregulation of miR-126-5p and the downregulation of zinc finger E-box-binding homeobox 2 to suppress cell viability and metastasis (73). In another study, paeonol repressed TNF-α, IL-6, IL-1β and TGF-β in the inhibition of non-small-cell lung cancer cell proliferation, invasion and migration by suppressing the STAT3/NF-κB pathway (74). Lei *et al* (75) reported that paeonol improved radiation-induced apoptosis via the suppression of the PI3K/AKT pathway to decrease downstream COX-2 and survivin. Recently, Zhou *et al* (76) investigated the effects of paeonol on osteosarcoma. They reported that the antitumor mechanisms were associated with the suppression of the invasion and migration potential, as well as the suppression of TLR4 to inhibit the activation of the MAPK/NF-κB downstream signaling pathway (76). In ovarian cancer, paeonol has been shown to suppress Akt/mTOR signaling by upregulating autophagy (77). Han *et al* (78) demonstrated that paeonol activated PTEN to suppress P-glycoprotein, multidrug resistant mutation 1 and metadherin to overturn resistant ovarian cancer. In another study, paeonol induced apoptosis via the upregulation of caspase-3 and caspase-9, and decreased p-Akt and p-GSK-3β signaling to inhibit ovarian cell proliferation (79). Zhou *et al* (80) demonstrated that paeonol increased the responsiveness of ovarian cancer cells to radiotherapy-induced apoptosis by inhibiting the PI3K/Akt/phosphatase, VEGF and HIF-1α pathways. In another study, cell viability, apoptosis, caspase-3 and survivin levels were evaluated by MTT assay, flow cytometry and Hoechst staining, and western blot analysis, respectively (81). The results revealed that cell viability was decreased, and apoptosis induced. Additionally, caspase-3 expression was upregulated and survivin protein expression was downregulated (81). In another study, paeonol administered *in vivo* and *in vitro* to human prostate cancer decreased tumor cell proliferation and suppressed tumor growth (82). In the process, the drug induced apoptosis which resulted in the upregulation of caspase-3, caspase-8, and caspase-9 levels and the downregulation of Bcl-2 with an enhanced Bax expression. Moreover, the activated PI3K/Akt pathway was inhibited. These findings suggest that the drug induces apoptosis by activating both the extrinsic and intrinsic pathways, and via the suppression PI3K/Akt pathway to inhibit tumor cell growth (82). Another study demonstrated that paeonol administration increased miR-141 by downregulating the protein kinase Cδ and c-Src pathway in the suppression of metastatic chondrosarcoma (83). Paeonol has been reported to possess anti-metastatic properties in pancreatic cancer. Researchers employed cell scratch-wound healing assay and Boyden chamber invasion assay to evaluate the migration and invasion abilities (84). In addition, the RNA and protein levels of E-cadherin, N-cadherin, vimentin and TGF-β1/Smad pathway were determined using RT-qPCR and western blot analysis. The findings revealed that paeonol suppressed epithelial-mesenchymal-transition by enhancing E-cadherin and reducing N-cadherin and vimentin. The expression levels of TGF-β1, p-Smad2/Smad2 and p-Smad3/Smad3 were also decreased to suppress the TGF-β1/Smad pathway (84). Paeonol induces apoptosis via the mitochondrial pathway and inhibits the PI3K/Akt pathway in the suppression of cervical cancer (85). It has been demonstrated that paeonol decreases 5-lipoxygenase to promote apoptosis in the inhibition of cervical cancer migration and invasion (33). It has been documented that paeonol suppresses cell proliferation, induces apoptosis and inhibits pro-inflammatory cytokine-mediated NF-κB and STAT3 signaling in the inhibition of melanoma metastasis (25). A previous study on renal cell carcinoma revealed that paeonol reduced the Bcl-2/Bax ratio in the induction of apoptosis and downregulated VEGFA to inhibit cell proliferation, invasion and metastasis (86). In another *in vitro* and *in vivo* study, paeonol downregulated miR-21-5p to upregulate KLF6 to promote apoptosis and inhibit the proliferation, migration and invasion of hepatocellular carcinoma cells (87). Li *et al* (88) evaluated the apoptotic activity of paeonol in hepatocellular carcinoma and reported that the drug decreased NF-κB p65/50 and protein apoptosis inhibitor-5 in the promotion of apoptosis and suppressed NF-κB signaling pathway. Fan *et al* (89) demonstrated the ability of paeonol to overturn endoplasmic reticulum stress-promoted resistance to doxorubicin by downregulating COX-2 to inactivate the PI3K/AKT/CHOP pathway. Paeonol has been reported to suppress tumor growth and promotes apoptosis by inhibiting mutant p53 and COX-2 and upregulating caspase-9 in 7,12-dimethylbenz(a)anthracene-induced oral carcinogenesis (90). The mechanism of paeonol in tumor immunity revealed that the drug downregulates PD1 via upregulating miR-139-5p to suppress melanoma growth (91). Research on aminothiazole-paeonol derivatives has shown that these compounds exhibit higher potency against AGS (gastric) and HT-29 (colorectal) human cancer cell lines compared to 5-fluorouracil (5-FU), a commonly used chemotherapeutic agent. Additionally, these derivatives demonstrated lower cytotoxicity towards normal cells, suggesting a potentially improved therapeutic index (92). Key signaling pathways and mediators downregulated by paeonol are illustrated in Fig. 1. The chemopreventive mechanisms mentioned above are presented in Table III.

The chemopreventive capacity of paeonol relies on its multifaceted anticancer mechanisms by directly inhibiting cell growth and proliferation, inducing apoptosis, suppressing invasion and metastasis (MMP-2 and-9, VEGF and HIF-1α), and inhibiting the TLR4/NF-κB/STAT3/MAPK/PI3K/AKT/CHOP pathways to inhibit cancer development and progression. Moreover, the drug has demonstrated potential for combination therapy and synergy with natural compounds, by improving their potency while potentially reducing their harmful effects. Additionally, paeonol reduces drug resistance in breast cancer and ovarian cancer, by decreasing drug efflux transporters and overturning resistance-associated signaling pathways.

Whilst there are data demonstrating the anticancer potential of paeonol, a search through published literature revealed limited *in vitro* and *in vivo* information regarding the toxicity of the compound on normal cells and organs. On the contrary, there is a huge volume of data on its ability to protect against chemical induced toxicity (26,35,62). Subchronic toxicity studies in male and female laboratory rats showed no major safety concerns (64,93). This attests to the potential of paeonol as a safe alternative agent for immunomodulation.

## 8. Clinical relevance and therapeutic effects on the NF-κB, MAPK and PI3K/AKT pathways

The NF-κB signaling pathway is crucial in regulating immune responses and inflammation. Paeonol has been shown to inhibit this pathway, thereby reducing the expression of pro-inflammatory cytokines and promoting apoptosis in cancer cells. For instance, studies have demonstrated that paeonol can effectively decrease NF-κB activation in hepatocellular carcinoma cells, leading to enhanced apoptosis and reduced cell proliferation (88,94). The inhibition of NF-κB in preclinical models by paeonol has been associated with decreased tumor growth and improved outcomes in inflammatory conditions such as ulcerative colitis (94,95).

The MAPK pathway is essential in cellular response to stress and growth. The regulation of MAPK by paeonol has been demonstrated to be linked to suppressed cell migration and invasion in lung cancer models, indicating its significant anti-metastatic potential (74,94). The dysregulation of the MAPK cascade by paeonol, not only inhibits cancer growth, but also alleviates inflammation in patients with chronic inflammatory diseases or cancer (74,96).

The PI3K/AKT pathway modulates cell survival, proliferation and metabolism, and its hyperactivation is commonly associated with cancer progression and resistance to apoptosis. Paeonol has demonstrated the ability to inhibit PI3K/AKT signaling to induce apoptosis and suppress tumor growth (68,75,82,94,97). The PI3K/AKT signaling modulation by paeonol has been linked to an improved sensitivity to chemotherapeutic agents and diminished adverse effects from treatment, suggesting its potential as an adjunct therapy in distinct populations (66,94,97).

## 9. Conclusion and future perspectives

Paeonol, a bioactive component of Cortex moutan has shown promise as a naturally occurring pharmacological agent for suppressing immunological events in a variety of human diseases. The mechanism is attributed to its multifactorial activities, including immunomodulatory effects, anti-inflammatory effects and antioxidant properties. Overall, paeonol decreases IL-1β expression to repress several inflammatory mediators, such as NO, iNOS, COX2, PEG2 in the inhibition of the NLRP3 inflammasome, NF-κB, MAPK and TLR4 pathways to provide multiple levels immunosuppressive effects of immune-related diseases. Current research demonstrates the anti-inflammatory properties of paeonol through the suppression of cytokines and the modulation of immune pathways. However, further investigations are required to focus on RNA-seq analyses to identify global gene expression changes in response to paeonol. Moreover, integrating multi-omics approaches (e.g., transcriptomics, proteomics and metabolomics) to build a systems-level understanding of the actions of paeonol could ultimately lead to the development of improved therapeutic strategies for conditions characterized by inflammation. The present review suggests that paeonol may serve as an alternative immunomodulator to conventional therapeutic agents; however, its probable negative effects in clinical applications need to be assessed. The repression of the NF-κB and MAPK pathways may not only reduce pro-inflammatory determinants, but may also attenuate the induction of certain key factors in the immune response. Thus, the effects of paeonol on different cytokines, inflammatory mediators and immune responses signaling pathways need to be explored. There are limited reports available of its application in relation to the cells of the immune system, effector mechanisms and phagocytosis, and thus, further studies are warranted.

As regards cancers, it has been reported that paeonol directly suppresses the proliferation and growth of several cancer cells, arrests the cancer cell cycle, induces apoptosis, and prevents invasion and metastasis. Furthermore, the drug inhibits cancer cell growth and proliferation through the suppression of the VEGF, HIF-1α, TLR4/ NF-κB/STAT3/MAPK/PI3K/AKT/CHOP pathways. Despite promising preclinical findings, there is a notable lack of clinical trial studies evaluating the efficacy and safety of paeonol treatment for human diseases. This underscores the need to conduct large-scale, multicenter clinical trials to validate preclinical findings in conditions such as asthma, atopic dermatitis, and allergic rhinitis, kidney injury and hepatotoxicity, breast cancer, gastric cancer and ovarian cancer to assess the therapeutic efficacy and safety of this drug in diverse patient populations. Moreover, exploring the synergistic effects of paeonol in combination with paclitaxel, epirubicin, doxorubicin, and apatinib in cancer treatment is warranted.

## Figures and Tables

**Table I:** *In vitro* and *in vivo* immunosuppressive effects of paeonol.

Authors, year of publication	Study type	Mechanism of action	(Refs.)
Yuan *et al*, 2022	*In vivo* and *in vitro*	Downregulation of IL-1β, IL-6 and ROS, and the suppression of M1 macrophage polarization and NLRP3 inflammasome	(21)
Pan *et al*, 2022	*In vivo* and *in vitro*	Suppresses the HIF-1α, IL-17A and IL-23 protein level	(34)
Kang *et al*, 2022	*In vivo* and *in vitro*	Downregulates NO, COX-2 and IL-6, IL-1β, TNF-α, IL-4 and NF-κB and MAPK	(30)
Miao *et al*, 2020	*In vivo* and *in vitro*	Restricts HMGB1 to the nucleus and promotes focal adhesion kinase; stimulates the development of pseudopods to upregulate phagocytosis	(32)
Sun *et al*, 2020	*In vivo* and *in vitro*	Suppresses miR-155/JAK1-STAT1 and downregulates F4/80 and CD86, IL-6 and TNF-α	(44)
Meng *et al*, 2019	*In vivo* and *in vitro*	Regulates the Th1/Th2 ratio by downregulating IgE, IL-4, histamine, IL-13, IL-31, TNF-α and p38/ERK/MAPK	(31)
Liu *et al*, 2018	*In vivo* and *in vitro*	Suppresses NF-κB, IL-6 and TNF-α through the adenosine 5'-monophosphate-activated protein kinase (AMPK) and glycogen synthase kinase-3 (GSK-3) pathways	(29)
Liu *et al*, 2018	*In vivo* and *in vitro*	Upregulates miR-223, but reduces STAT3, p-STAT3, IL-1β, IL-6, ICAM-1, VCAM-1 expression	(45)
Lou *et al*, 2017	*In vivo* and *in vitro*	Downregulates NO, INOS, COX-2, MMP-1, MMP-3, MMP-13, and inhibits NF-κB, PI3K and AKT	(46)
Meng *et al*, 2017	*In vivo* and *in vitro*	Inhibits IL-23 and downregulates dendritic cells expressing MHCII, CD80 and CD86	(17)
Fan *et al*, 2016	*In vivo* and *in vitro*	Represses pro-inflammatory cytokines and upregulates anti-inflammatory cytokines; inhibits IκBα and IKKβ, NF-κB p65, and restricts NF-κB p65 DNA-binding activity as well as TLR4-NF-κB	(18)
Wu *et al*, 2016	*In vivo* and *in vitro*	Suppresses the PI3K/Akt/NF-kB pathway.	(35)
Lin *et al*, 2015	*In vivo* and *in vitro*	Regulates AMPK-α and GSK 3α/β to suppress NO, iNOS, COX-2, ROS and STAT3 as well as p38 pathways	(47)
Liu *et al*, 2014	*In vivo* and *in vitro*	Inhibits MAPKs/NF-κB signaling pathways.	(26)
Chen *et al*, 2014	*In vivo* and *in vitro*	Downregulates TNF-α, IL-1β, IL-6, upregulates IL-10 via the deactivation of IκBα, ERK1/2, JNK, and p38 MAPK	(48)

IL, interleukin; ROS, reactive oxygen species; NO, nitric oxide; COX-2, cyclooxygenase 2; VCAM-1, vascular cell adhesion molecule 1; ICAM-1, intercellular adhesion molecule 1; MMP, matrix metalloproteinase.

**Table II:** *In vitro* or *in vivo* immunosuppressive effects of paeonol.

Authors, year of publication	Study type	Mechanism of action	(Refs.)
Niu *et al*, 2024	*In vitro*	Regulates M1/M2 polarization of macrophages	(49)
Chen *et al*, 2022	*In vitro*	Suppresses IL-1β and inhibits NLRP3, NF-κB, and MAPK activated pathways	(52)
He *et al*, 2016	*In vitro*	Represses NO, IL-1β and PGE2, as well as the expression of iNOS and COX-2; reduces TLR4, MyD88, IRAK4, TNFR-associated factor 6 (TRAF6), p-IkB-α, and NF-κB p65, as well as p-p38, p-JNK, and p-ERK	(53)
Ping *et al*, 2014	*In vitro*	Upregulates NO, iNOS, downregulates ROS, TNF-α, IL-1β, IL-6 and MCP-1 through the RAGE-, CD36-, SR-A- and SR-B1-mediated pathway	(28)
Li *et al*, 2023	*In vivo*	Modulating microglial M1 and M2 polarization via the RhoA/p38MAPK pathway	(54)
Ge *et al*, 2021	*In vivo*	Decreases the dectin-1/NF-κB pathway together with TLR2 and TLR4	(27)
Adki *et al*, 2021	*In vivo*	Suppresses NF-κB and MCP-1	(55)
Wu *et al*, 2020	*In vivo*	Suppresses the dectin-1/IL-1β signaling pathway	(56)
Wu *et al*, 2019	*In vivo*	Inhibits TGF-β/Smad3 and downregulates IL-6 and TNF-α	(57)
Li *et al*, 2019	*In vivo*	Upregulates Nrf2 to downregulate NF-κB/NFATc1	(58)
Tang *et al*, 2018	*In vivo*	Downregulates IFN-γ and upregulates IL-4, suppresses TLR4/NF-κB and MAPK pathway	(59)
Gong *et al*, 2017	*In vivo*	Suppresses iNOS, nitric oxide, COX-2, prostaglandin E2 and inhibitsNF-κB, and MAPK pathways as well as decreased hepatocyte apoptosis	(60)
Zong *et al*, 2017	*In vivo*	Decreases IL-17 and IL-6 and upregulates TGF-β1	(61)
Ding *et al*, 2016	*In vivo*	Suppresses c-Jun N-terminal kinase and decreases TNF-α, MCP-1, IL-1β, IL-6 and inhibits IKKα/β, IκBα and p65 phosphorylation	(62)
Liao *et al*, 2016	*In vivo*	Represses TLR2, TLR4, Iba1-, NF-κB- (P50-), and IL-1β and inhibits apoptosis	(63)
Lee *et al*, 2013	*In vivo*	Downregulates TNF-α, IL-1β and nitric oxide	(64)

IL, interleukin; iNOS, inducible nitric oxide synthase; COX-2, cyclooxygenase 2; TLR, Toll-like receptor; ROS, reactive oxygen species; PGE2, prostaglandin E2.

**Table III:** *In vitro* and *in vivo* chemopreventive effects of paeonol.

Authors, year of publication	Study type	Mechanism of action	(Refs.)
Saahene *et al*, 2018	*In vitro*	Regulates CXCL4/CXCR3-B signal to downregulate heme oxygenase (HO-1) and nuclear factor E2-related factor 2 (Nrf2) to upregulate BACH1 to induce apoptosis in breast cancer	(14)
Wu *et al*, 2016	*In vivo* and *in vitro*	Induces apoptosis via activation of PARP, Bax, caspase 3 and suppresses MAPKs/NF-κB pathways in breast cancer	(35)
Zhang *et al*, 2015	*In vitro*	Decreases SET, protein phosphatase 2A and phosphatidylinositol 3-kinase (PI3K)/Akt pathway in breast cancer	(66)
Cai *et al*, 2014	*In vitro*	Decreases P-glycoprotein, multidrug resistance associated protein 1, and breast cancer resistance protein to suppress transgelin 2 resistance	(67)
Qu *et al*, 2014	*In vitro*	Downregulates Bcl-2, upregulates Bax, caspase-8 and caspase-3 to induce breast cancer apoptosis	(24)
Zhang *et al*, 2021	*In vivo* and *in vitro*	Decreases Bcl-2/Bax ratio upregulates caspase 3 and suppresses PI3K/AKT pathway in bladder cancer	(68)
Liu *et al*, 2020	*In vitro*	Induces apoptosis, arrest the cell cycle at G0/G1-phase, downregulates β-catenin, cyclin D1, survivin and c-Myc to suppress Wnt/β-catenin pathway in colorectal cancer	(69)
Li *et al*, 2014	*In vivo* and *in vitro*	Decreases cyclooxygenase-2 and prostaglandin E2 and decreased Bcl-2 and upregulates Bax, caspase 3 and caspase 9 in colorectal cancer	(23)
Li *et al*, 2013	*In vitro*	Induces apoptosis, arrests the cell cycle at the G1 to S transition phase; upregulation of intracellular calcium concentration and RUNX3 in colon cancer	(70)
Li *et al*, 2022	*In vitro*	Downregulation of LINC00665 and MAPK1, upregulating miR-665 in gastric cancer	(71)
Fu *et al*, 2018	*In vitro*	Inhibits NF-κB by decreasing ERBB2 and induces apoptosis gastric cancer	(72)
Lyu *et al*, 2017	*In vitro*	Downregulation of MMP-2 and MMP-9 in gastric cancer	(22)
Lv *et al*, 2022	*In vitro*	Upregulates miR-126-5p and downregulates ZEB2 to suppress cell viability and metastasis in lung cancer	(73)
Zhang *et al*, 2020	*In vivo* and *in vitro*	Inhibits TNF-α, IL-6, IL-1β, TGF-β and suppressed STAT3/NF-κB pathways in lung cancer	(74)
Lei *et al*, 2013	*In vitro*	Enhances apoptosis, suppression of PI3K/AKT pathway and decreased cyclooxygenase-2 and survivin in lung cancer	(75)
Zhou *et al*, 2020	*In vivo* and *in vitro*	Suppresses TLR4 to inhibit MAPK/NF-κB to prevent invasion and migration and induces apoptosis in osteosarcoma	(76)
Gao *et al*, 2019	*In vivo* and *in vitro*	Suppresses Akt/mTOR signal by upregulating autophagy in ovarian cancer	(77)
Han *et al*, 2018	*In vitro*	Upregulation of PTEN suppresses P-glycoprotein, multidrug resistant mutation 1 and metadherin in ovarian cancer	(78)
Li *et al*, 2017	*In vitro*	Upregulates caspase-3 and caspase-9 to induces apoptosis and decreased phosphorylated-Akt and phosphorylated-GSK-3β in ovarian cancer	(79)
Zhou *et al*, 2017	*In vitro*	Inhibits P13K/Akt, VEGF and HIF-1α pathways via radiation induced apoptosis in ovarian cancer	(80)
Yin *et al*, 2013	*In vitro*	Upregulates caspase-3 and downregulate of survivin to induce apoptosis in ovarian cancer	(81)
Xu *et al*, 2017	*In vivo* and *in vitro*	Upregulates caspase-3, caspase-8, and caspase-9 events and downregulated Bcl-2 with an enhanced Bax; inhibits P13K/Akt pathway in prostate cancer	(82)
Horng *et al*, 2014	*In vitro*	Increases miR-141 and downregulated protein kinase C (PKC) δ and c-Src pathways in chondrosarcoma	(83)
Cheng *et al*, 2020	*In vitro*	Increases E-cadherin and reduced N-cadherin and vimentin; decreased TGF-β1, p-Smad2/Smad2 and p-Smad3/Smad3 in pancreatic cancer	(84)
Du *et al*, 2021	*In vitro*	Downregulates Bcl-2/Bax proportion, upregulated caspase-3 by inhibiting P13K/Akt pathway in cervical cancer	(85)
Sheng *et al*, 2021	*In vitro*	Downregulates 5-lipoxygenase to promote apoptosis to prevent invasion and migration in cervical cancer.	(33)
Zhang *et al*, 2015	*In vivo* and *in vitro*	Induces apoptosis and inhibits proinflammatory cytokines mediated NF-κB and STAT3 signaling to prevent melanoma metastasis	(25)
Chen *et al*, 2022	*In vitro*	Increases Bax decreased Bcl-2 and downregulated VEGFA to prevent invasion and metastasis in renal cell carcinoma	(86)
Cai *et al*, 2020	*In vivo* and *in vitro*	Downregulates miR-21-5p to upregulate KLF6 in the promotion of apoptosis and inhibited cell proliferation, migration and invasion of hepatocellular carcinoma.	(87)
Li *et al*, 2019	*In vitro*	Decreases NF-κB p65/50 and protein apoptosis inhibitor-5 in the induction of apoptosis and suppressed NF-κB pathway in HCC	(88)
Fan *et al*, 2013	*In vitro*	Downregulates COX-2 to inactivate the PI3K/AKT/CHOP pathway in hepatocellular carcinoma.	(89)
Ramachandhiran *et al*, 2018	*In vivo*	Promotes apoptosis by inhibiting mutant p53 and COX-2 and upregulating caspase-9 in oral carcinogenesis	(90)

IL, interleukin; iNOS, inducible nitric oxide synthase; COX-2, cyclooxygenase 2; TLR, Toll-like receptor; ROS, reactive oxygen species; PGE2, prostaglandin E2.

## Data Availability

Not applicable.
